# Comparative effects of walnut, apricot, and almond shell biochars on *in vitro* rumen fermentation and methane production

**DOI:** 10.1016/j.vas.2026.100858

**Published:** 2026-09-08

**Authors:** Mehri Montazerharzand, Hamid Paya, Akbar Taghizadeh, Ali Hosseinkhani, Mohammad Ramin

**Affiliations:** a Department of Animal Science, Agricultural Faculty, University of Tabriz, Tabriz, Iran; b Department of Applied Animal Science and Welfare, Swedish University of Agricultural Sciences, Umeå, 90183, Sweden

**Keywords:** Biochar, Methane mitigation, Volatile fatty acids, Protozoa, Dry matter degradability

## Abstract

An alternative strategy to reduce methane (CH₄) emissions from livestock is biochar, a porous carbonaceous material produced through the pyrolysis of biomass. This study aimed to evaluate the effects of biochars derived from walnut shell, almond shell, and apricot shell on rumen fermentation and CH₄ production under *in vitro* conditions. The experiment used a completely randomized design in an *in vitro* fermentation system with four treatments: a control diet without biochar and the control diet supplemented with 1% (based on total substrate dry matter) walnut, almond, or apricot shell biochar. Gas production was measured at multiple time points, and fermentation parameters were analyzed after 24 h of incubation. Walnut shell biochar increased gas production throughout the incubation period, whereas apricot shell biochar decreased it compared to the control (P < 0.05). While pH remained unaffected, apricot shell biochar significantly reduced ammonia nitrogen (NH₃-N) (P < 0.05). Furthermore, apricot and almond shell biochars significantly reduced protozoa counts (P < 0.05). Notably, walnut shell biochar significantly increased total volatile fatty acids (VFA) and propionate concentrations (P < 0.05), and enhanced *in vitro* dry matter degradability (DMD) (72.3% *vs.* 65.3% in the control) (P < 0.05). All biochar treatments significantly reduced CH₄ production (P < 0.05), with the greatest reduction observed with walnut shell biochar (77.2 vs. 90.9 mL/g DM; approximately 15% reduction). Overall, walnut shell biochar showed the most favorable effects by enhancing *in vitro* DMD, increasing total VFA and propionate concentrations, and achieving the greatest reduction in CH₄ production.

## 1 Introduction

Ruminants possess a unique ability to convert fibrous plant materials, indigestible to humans, into high-quality protein sources like milk and meat (Broderick, 2018). During this process, fermentation of carbohydrates generates metabolic hydrogen, which is utilized by methanogenic archaea to reduce CO_2_ and produce methane (CH_4_) (Wang et al., 2023).

Methane emissions are a significant contributor to global greenhouse gas (GHG) emissions, playing a critical role in climate change mitigation efforts. Among the primary GHGs, CH_4_ possesses a substantially higher global warming potential than carbon dioxide over shorter time scales (Jeong et al., 2022). In ruminants, rumen fermentation is a major source of CH_4_ emissions. Furthermore, methanogenesis during rumen digestion results in an energy loss of 2–12% of the animal’s gross energy intake (Johnson & Johnson, 1995; Kumara et al., 2014). Therefore, effective mitigation strategies are needed to reduce enteric CH_4_ emissions while maintaining animal productivity.

Globally, agricultural residues are produced in vast quantities each year, creating challenges for efficient disposal. Traditional processing methods, such as composting and open burning, are associated with air pollution and low resource utilization efficiency (Alhazmi & Loy, 2021). The circular economy transforms agricultural waste into a valuable resource; by closing the loop through recycling and reprocessing, it moves beyond the linear ‘extract-produce-dispose’ model to ultimately achieve optimized resource efficiency and promote agricultural sustainability (Barros et al., 2020). Through thermochemical processing methods such as pyrolysis, these residues can be transformed into value added products, including biochar, which has emerged as a key material for environmental and agricultural applications due to its sustainable characteristics (Li et al., 2022; Seow et al., 2022).

The primary objective of ruminant nutritionists is to optimize rumen metabolic efficiency and enhance animal productivity. Traditionally, a variety of feed additives, including antibiotics, ionophores, and CH_4_ inhibitors, have been incorporated into ruminant diets. However, growing concerns about chemical residues in animal derived food products and the emergence of bacterial resistance have intensified public demand for safer and high quality food (Han et al., 2024). In response to these concerns, researchers have focused on identifying natural alternatives to regulate rumen fermentation and improve livestock production.

Among these natural alternatives, carbon-based compounds such as biochar have emerged as promising candidates. Biochar is a porous, carbon rich material generated through the thermal decomposition of diverse biomass sources at temperatures between 400 and 900 °C in an oxygen-limited environment, typically under a flow of nitrogen or helium (Wang et al., 2020). Biochar is widely used in environmental management and agriculture, and recent studies have explored its application as a feed additive in ruminant nutrition. Supplementation of biochar at levels of 1–3% of livestock dry matter intake (DMI) has shown potential to improve rumen fermentation and reduce CH_4_ production (Burezq & Khalil, 2025; Saleem et al., 2018).

In this context, recent studies have highlighted the potential of various agricultural residues as sustainable feed additives, demonstrating their effectiveness in modulating ruminal fermentation and mitigating CH_4_ production (Dey et al., 2025; Wanapat et al., 2024). Their potential impact on rumen fermentation has attracted significant interest from nutritionists, paving the way for innovative approaches in livestock nutrition research.

These effects are attributed to the unique physicochemical properties of biochar, including high porosity, gas adsorption capacity, redox activity, and provision of habitats for microbial colonization. Such characteristics may influence rumen fermentation dynamics by modifying microbial activity and hydrogen utilization, thereby contributing to reduced CH_4_ production (Teoh et al., 2019). However, the quality and physicochemical characteristics of biochar are strongly influenced by biomass source as well as pyrolysis conditions such as residence time and temperature. The hard shells of walnut, almond, and apricot are lignocellulosic residues with relatively high lignin content and rigid structures, which may yield biochars with distinct porosity, surface area, carbon content, and adsorption capacity (Lao & Mbega, 2020). These anticipated differences were confirmed by the measured physicochemical properties of the produced biochars (Table 2). In addition, these hard shells are abundantly available agricultural by‑products in the study region, making them practical and sustainable feedstocks for biochar production. Therefore, differences among these biomass sources may lead to variable effects on rumen fermentation and CH_4_ production.

However, comparative information on biochars derived from different hard shell residues and their effects on rumen fermentation and CH_4_ production remains limited. To our knowledge, a direct comparison of biochar produced from walnut, almond, and apricot shells under similar experimental conditions has not been widely reported. Based on these considerations, it was hypothesized that biochars derived from walnut, almond, and apricot shells may differ in their physicochemical characteristics and consequently exert different effects on dry matter degradability (DMD), rumen fermentation characteristics, and CH_4_ production. Therefore, the objective of this study was to comparatively evaluate the effects of biochar derived from walnut, almond, and apricot shells on DMD, rumen fermentation characteristics, and CH_4_ production under *in vitro* conditions.

## 2 Methods

### 2.1 Sample preparation and biochar production

Biochar preparation followed the method described by Montazerharzand et al. (2025). Walnut, almond, and apricot shell wastes were collected (local orchards in Tabriz, Iran) and washed with water to remove external impurities, then dried at room temperature for 3 days. To produce biochars, the samples were ground using a grinder with a 5 mm sieve, then placed in an electric furnace under argon gas (5 L per minute) at a temperature of 500 °C with a heating rate of 5 °C per minute for 4 h. After the furnace temperature gradually returned to room temperature, the biochars were removed from the furnace and stored in sealed plastic containers at room temperature. The mass yield of the biochar was calculated as the percentage of the biochar produced relative to the initial weight of the raw material. The ash content was measured by burning the biochar at 750 °C for 6 h (ASTM, 2007).

The elemental composition (carbon (C), nitrogen (N), hydrogen (H), and sulfur (S) (CHNS)) of the biochar was analyzed using an elemental analyzer (Costech-ECS 4010-USA). The oxygen (O) content of the samples was calculated by subtracting the sum of C, N, H, S, and ash from 100. The surface area of the biochar samples was determined using nitrogen adsorption/desorption isotherms at 77 K with a surface area analyzer (Belsorp-Mini II, Japan).

### 2.2 Animals and treatments

Rumen fluid for this study was obtained from two fistulated sheep. Four dietary treatments were evaluated: (1) A control diet without biochar, (2) A control diet supplemented with 1% walnut shell biochar, (3) A control diet supplemented with 1% almond shell biochar and (4) A control diet supplemented with 1% apricot shell biochar. The biochars were added as a top dressing to the control diet, which consisted of 40% forage and 60% concentrate, at 1% of the total substrate dry matter. It should be noted that the incubated diet differed from the maintenance diet of the donor animals. The 1% inclusion level was selected based on previous studies showing favorable effects of biochar at similar inclusion levels. Prior to the incubation procedures, it should be noted that the entire experiment was conducted in a single experimental run, and all replicates were treated as independent experimental units.

### 2.3 Feed chemical composition analysis

For the chemical composition analysis of the feed, samples were dried at 60 °C for 48 h and ground using a 2-mm sieve. The feed's dry matter (DM), ash, crude fat, and crude protein contents were analyzed according to the methods outlined by AOAC (2005). Acid detergent fiber (ADF) and neutral detergent fiber (NDF) were determined following the method of Van Soest et al. (1991), without the use of heat-stable alpha-amylase or sodium sulfite. The experimental diets chemical composition were identical to those reported previously (Montazerharzand et al., 2025). The chemical composition of the diets is presented in Table 1 for clarity.

**Table 1:** Chemical composition and ingredients of experimental diets (adapted from Montazerharzand et al., 2025).

Ingredients (%)		Chemical composition (%)	
Alfalfa	30	Dry matter	95.3
Wheat straw	10	Crude protein	16.8
Barley grain	30	Ash	7.68
Wheat bran	20.7	Ether extracts	1.65
Soybean meal	7.8	Neutral detergent fiber	38.1
Salt	0.5	Acid detergent fiber	20
Sodium bicarbonate	0.5	Metabolizable energy (Mcal/kg)	2.02
Minerals and vitamins supplement	0.5		

### 2.4 Gas production and estimated parameters

The gas production technique described by Fedorah and Hrudey (1983) was employed to assess the impact of biochars derived from walnut, almond, and apricot shells on the fermentability of the control diet. For the experiment, 300 mg of the ground control diet (2-mm sieve) and 1% of the respective biochars were added to sterilized 50 mL glass vials. Each treatment was initially incubated in seven replicates; however, two replicates were identified as outliers based on statistical evaluation and excluded from the analysis, resulting in five replicates per treatment. (All vials were placed in the same incubator; however, each vial was considered an independent experimental unit). Male Ghezel sheep (approximately 48 kg body weight) fitted with rumen fistulas were used as rumen fluid donors. The animals were fed a maintenance diet consisting of 60% forage and 40% concentrate. Rumen fluid (approximately 400 mL from each fistulated sheep) was collected from two fistulated sheep approximately two hours after the morning feeding, filtered through a four-layer cheesecloth, and transferred to the laboratory in a flask maintained at 39 °C. For each vial containing the experimental treatments, 20 mL of a mixture of rumen fluid and McDougall’s buffer (containing 9.8 g NaHCO₃, 9.3 g Na₂HPO₄·12H₂O, 0.47 g NaCl, 0.57 g KCl, 0.04 g CaCl₂ (*anhydrous*) and 0.06 g MgCl₂ (*anhydrous*) according to McDougall, 1948; supplemented with 5 g glucose and 0.5 g L-Cysteine added on the day of the experiment; final volume adjusted to 1 L with distilled water) was added at a ratio of 1 part rumen fluid to 2 parts buffer. The vials were sealed with rubber stoppers and secured with aluminum caps. To correct gas production, five blank vials containing only 20 mL of buffered rumen fluid mixture were prepared. All vials were incubated in a shaking incubator set at 70 rpm and 39 °C. Gas production was recorded at 2, 4, 6, 8, 12, 24, 36, 48, 72, and 96 h of incubation (Gas volume was measured repeatedly from the same incubation vials without opening them).

The gas volume produced at each incubation time was corrected based on the sample weight using the following equation:

$$V=\frac{\text{Vt}-\text{Vb}}{W}\times 1000$$

where: V = corrected gas volume (mL/g DM), Vt = gas volume produced in vials containing the feed sample (mL), Vb = gas volume produced in blank vials without feed sample (mL), and W = weight of the feed sample (mg DM).

Gas production parameters were calculated using the exponential model proposed by Ørskov and McDonald (1979): $P=A(1-e^{-\text{ct}})$, where P is the gas production at time, A is the potential gas production (A = a + b), a represents gas production from the rapidly soluble fraction, b represents gas production from the insoluble but fermentable fraction, c is the fractional rate constant of gas production, t is the incubation time, and *e* is a constant called Euler’s number, approximately equal to 2.718. These parameters were analyzed using the Fitcurve software.

Metabolizable energy (ME), net energy for lactation (NE_l_), and organic matter digestibility (OMD) of the samples were calculated using the equations developed by Menke et al. (1979) and Menke and Steingass (1988). Short-chain fatty acid (SCFA) was estimated based on the equations proposed by Getachew (2002).

$$\begin{array}{c} \mathrm{ME}\left(\mathrm{MJ}/\mathrm{KgDM}\right)=2.2+\left(0.136\times \mathrm{GP}\right)+\left(0.057\times \mathrm{CP}\right)+\left(0.002859\times CF^{2}\right)\\ NE_{L}\left(\mathrm{MJ}/\mathrm{KgDM}\right)=\left(0.101\times \mathrm{GP}\right)+\left(0.051\times \mathrm{CP}\right)+\left(0.11\times \mathrm{CF}\right)\\ \begin{array}{c} \mathrm{OMD}\left(\%\mathrm{DM}\right)=14.88+\left(0.8893\times \mathrm{GP}\right)+\left(0.448\times \mathrm{CP}\right)+\left(0.651\times \mathrm{CA}\right)\\ \mathrm{SCFA}\left(\mathrm{mmol}/200\;\mathrm{mgDM}\right)=\left(0.0222\times \mathrm{GP}\right)-0.00425 \end{array} \end{array}$$

In these equations, GP is 24 h net gas production (mL/200 mg DM); CP, CF and *CA* are crude protein, crude fat and crude ash (%DM), respectively.

### 2.5 Rumen ecosystem and methane production

After 24 h of incubation using the gas production technique, the liquid phase was sampled to estimate rumen fermentation parameters (three replicates per treatment), including pH, ammonia nitrogen (NH_3—_N), volatile fatty acids (VFAs), and protozoa counts (Separate vials were incubated for 24 h and opened for fermentation analysis.). The pH of the incubated liquid was measured immediately after opening the vials using a portable pH meter (Model AD11, Adwa Instruments, Hungary). For protozoa counting, the liquid was mixed with a formalin solution (prepared by dissolving 100 mL of 37% formaldehyde and 8.5 g of sodium chloride in distilled water to a final volume of 1 L) in a 1:4 ratio (1 part rumen fluid to 4 parts formalin) to halt microbial activity. Samples were stored in a refrigerator until analysis. During counting, a drop of each sample was placed on a Neubauer chamber, covered with a coverslip, and observed under a light microscope at 10 × magnification (Ivan et al., 2013). Total protozoa were counted without differentiation of genera. For the final calculation of protozoa concentration (cells/mL), the following formula was used:

$$Protozoa/mL=\left(N\;\times \;5\;\times 10^{3}\right)/0.1$$

where N is the total number of protozoa counted in the specified corner squares, 5 is the dilution factor (from the 1:4 rumen fluid to formalin dilution), and the denominator 0.1 represents the adjusted counted volume in mm^3^ used in the final calculation formula.

To quantify ammonia nitrogen, the liquid was mixed in a 5:1 ratio with 0.2 N hydrochloric acid (5 parts liquid to 1 part HCl). Volatile fatty acids were analyzed by mixing the liquid with 50% sulfuric acid (1:50 ratio; 1 part liquid to 50 parts H_2_SO_4_). Both types of samples were stored at −20 °C until further analysis. Ammonia nitrogen was measured spectrophotometrically at a wavelength of 630 nm using the method described by Broderick and Kang (1980). Volatile fatty acids were quantified using gas chromatography (Agilent 6890 N, USA), equipped with a flame ionization detector (FID). Injector and detector temperatures were set at 240 °C and 270 °C, respectively. The carrier gas was nitrogen at a flow rate of 1.8 mL/min. A DB-FFAP capillary column (J&W 123–3232, 30 m length, 320 μm internal diameter, 0.25 μm film thickness) was used. The column temperature was programmed to increase from 80 °C (held for 2 min) to 160 °C at a rate of 10 °C/min. The 2-Ethyl butyric acid served as the internal standard.

### 2.6 Methane production analysis

The effect of biochar on CH_4_ production was investigated using an *in vitro* method with glass syringes. For each treatment, 200 mg of feeds was combined with the respective biochar (three replicates per treatment). Then, 20 mL of the McDougall buffered rumen fluid mixture (1:2 ratio) was added to the syringes. The syringes were incubated at 39 °C for 24 h. Gas production was measured post-incubation, and gas samples were collected using 10-mL evacuated tubes. Methane production was analyzed via gas chromatography (multi-channel gas chromatograph with DSQ II mass spectrometer, AS2000 Autosampler and ThermoQuest controller) using the procedure described by Adejoro et al. (2020). The column, injector, and detector temperatures were set at 100 °C, 200 °C, and 250 °C, respectively, with helium as the carrier gas at a flow rate of 24 mL/min. CH_4_ concentration was calculated using a regression equation derived from pure CH_4_ standards of varying concentrations.

### 2.7 Dry matter degradability

The effect of biochar addition on feed degradability was assessed using the method described by Holden (1999). Half a gram of ground control feed (sieved through a 2-mm screen) was placed in nylon bags free of nitrogen and ash, which were then sealed. All *in vitro* degradability incubations were conducted in a single experimental run using pooled rumen inoculum obtained from two donor animals. For each treatment, four nylon bags were incubated simultaneously in a single digestion bottle containing 1400 mL of buffer solution and 400 mL of rumen fluid. The bottles were incubated at 39 °C for 48 h. After this initial incubation, 40 mL of 6 N hydrochloric acid and 8 *g* of pepsin were added to each bottle, and the incubation was extended for an additional 24 h at the same temperature. Upon completion of the digestion process, the nylon bags were rinsed thoroughly with cold water to remove residues, dried in an oven at 100 °C, and weighed. The results were used to calculate *in vitro* DMD.

### 2.8 Statistical analysis

The experimental data were analyzed using a completely randomized design (CRD) model. Analysis of Variance (ANOVA) was conducted via the SAS software (version 9.2) using the General Linear Model (GLM) procedure. Normality was evaluated using the Shapiro–Wilk test. Duncan’s multiple-range test was employed for mean comparisons among treatments for all measured variables. The statistical model used for the analysis was:

$$Y_{ij}=\mu +T_{i}+e_{ij}$$

Where: Y_ij_: Observed value, μ: Overall mean, T_i_: Effect of the treatment, e_ij_: Experimental error.

## 3 Results

### 3.1 Biochar yield, elemental analysis, and Brunauer-Emmett-Teller surface area

Table 2 presents the yield, elemental composition, and Brunauer-Emmett-Teller (BET) surface area of the biochars obtained from walnut, almond, and apricot shells. The biochar yields for walnut and apricot shells were similar (32.2% and 30.7%, respectively) but significantly different from that of almond shells (21.5%). The C content of the biochar samples was 84.5%, 93.2%, and 79.3% for walnut, almond, and apricot shells, respectively. The highest BET surface area was reported for walnut shell biochar (618 m²/g). This reported value is significantly different from the surface area of almond and apricot biochars (178 m²/g and 101 m²/g, respectively). The surface BET values were as follows: WS-B > Als-B > Aps-B.

**Table 2:** Characteristics of biochar made from walnut shell (Ws), almond shell (Als), apricot shell (Aps) at 500 °C.

	WS-B	Als-B	Aps-B
Mass yield (%)	32.2	21.5	30.7
Ash content (%)	2.40	1.25	1.05
Element analysis (wt%)			
Carbon	84.5	93.2	79.3
Hydrogen	1.3	0.42	1.91
Nitrogen	1.25	1.38	0.82
Sulphur	0.23	0.29	0.22
Oxygen	10.3	3.50	16.7
H/C	0.01	0.004	0.02
O/C	0.12	0.03	0.21
BET surface area (m^2^/g)	618	178	101

### 3.2 Gas production, gas production kinetic, and estimated parameters

The gas production values for treatments with biochars derived from walnut, almond, and apricot shells (added at a 1% inclusion level of DM) are presented in Table 3, Table 4. Gas production kinetic and estimated parameters (corrected to 200 mg of DM) are also shown. Gas production differed significantly among the experimental treatments during the incubation period (P < 0.05). At the early incubation times (2–8 h), walnut shell and almond shell biochars generally produced higher gas volumes than the other treatments, whereas apricot shell biochar consistently exhibited the lowest values. As incubation progressed (12–48 h), the differences among treatments became more pronounced, with walnut shell biochar maintaining the highest gas production throughout this period (P < 0.05). Gas production in the almond shell biochar treatment was generally comparable to that of the control, although slight variations above or below the control were observed at some incubation times. In contrast, apricot shell biochar remained associated with the lowest gas production values (P < 0.05). A similar pattern was observed at the later incubation times (72 and 96 h), where walnut shell biochar produced the greatest gas volume, while the control and almond shell biochar showed comparable values, and apricot shell biochar consistently produced the lowest gas production (P < 0.05).

**Table 3:** Effect of experimental treatments on gas production at different incubation times and kinetic parameters of gas production.

Experimental treatments*						
	Con	Con+WS-B	Con+Als-B	Con+Aps-B	SEM	*p*-value
**gas production (mL/g DM)**						
2	33.4^a^	35.4^a^	35.1^a^	30.2^b^	0.63	<0.001
4	67^b^	71.6^a^	71.4^a^	65.3^b^	0.72	<0.001
6	99.6^b^	104^a^	101 b	93.6^c^	0.97	<0.001
8	123^a^	125^a^	125^a^	117^b^	1.02	<0.001
12	146^b^	149^a^	147^a^^,^^b^	140^c^	1.02	<0.001
24	194^b^	200^a^	191^c^	180^d^	0.59	<0.001
36	217^b^	226^a^	214^b^	204^c^	1.21	<0.001
48	234^b^	244^a^	226^c^	218^d^	1.23	<0.001
72	247^b^	255^a^	245^b^	234^c^	0.68	<0.001
96	255^b^	261^a^	254^b^	238^c^	0.90	<0.001
**gas production kinetics**						
a (mL/g DM)	12.8^b^	14.9^a^^,^^b^	18.2^a^	12.7^b^	1.33	0.0346
b (mL/g DM)	235^a^	228^b^	226^b^	218^c^	1.67	<0.001
a + b (mL/g DM)	248^a^	243^a^	245^a^	231^b^	2.80	0.0024
c (mL/h)	0.06	0.07	0.06	0.07	0.002	0.5416

⁎ Experimental diets contained without biochar as a control (CON), walnut shell biochar (WS-B 1%), almond shell biochar (Als-B 1%), and apricot shell biochar (Aps-B 1%) (n = 5). Values within a row with different superscripts differ significantly at p < 0.05.

a : gas production from the rapidly soluble fraction.

b : gas production from the insoluble but fermentable fraction, a + b (A): potential gas production.

c : gas production rate (ml/h). SEM: Standard error of means.

**Table 4:** Effect of experimental treatments on gas production parameters and rumen and total tract disappearance of DM.

	Experimental treatments*					
	Con	Con+WS-B	Con+Als-B	Con+Aps-B	SEM	*p*-value
*Parameters*						
ME (MJ/ kg DM)	8.44^b^	8.61^a^	8.38^c^	8.16^d^	0.02	<0.001
NE_L_ (MJ/ kg DM)	4.96^b^	5.08^a^	4.91^c^	4.75^d^	0.01	<0.001
OMD (%)	61.9^b^	63.0^a^	61.5^c^	60.0^d^	0.13	<0.001
SCFA (mmol/ 200 mg DM)	0.85^b^	0.88^a^	0.84^c^	0.81^d^	0.003	<0.001
*Degradability (%)*						
Rumen	65.3^b^	72.3^a^	66.6^b^	67.7^b^	0.94	0.0012
Total tract	74.5^a^	75.6^a^	76.0^a^	71.0^b^	0.92	0.0099

⁎ Experimental diets contained without biochar as a control (CON), walnut shell biochar (WS-B 1%), almond shell biochar (Als-B 1%), and apricot shell biochar (Aps-B 1%) (n = 5). Values within a row with different superscripts differ significantly at p < 0.05. ME: metabolizable energy, NE_L_: net energy of lactation, OMD: organic matter digestibility, and SCFA: short-chain fatty acid.

The potential gas production for the different treatments (control, walnut, almond, and apricot shell biochars) was 248, 243, 245, and 231, respectively. Based on the comparison of means, apricot shell biochar showed a significantly lower value than the other treatments (P < 0.05), whereas no significant differences were observed among the control, walnut shell biochar, and almond shell biochar treatments. In addition, no significant differences were detected among treatments in terms of the gas production rate (P > 0.05).

The estimated values for ME, NE_L_, OMD, and SCFAs aligned with the trends observed in gas production, as these parameters are predicted from gas production using established equations. Walnut shell biochar, which enhanced total gas production, also yielded the highest values for these parameters (P < 0.05). Conversely, apricot shell biochar, which reduced total gas production, showed the lowest values for these parameters (P < 0.05).

### 3.3 Rumen ecosystem

The effects of biochars derived from walnut, almond, and apricot shells on ruminal parameters such as pH, protozoa population, ammonia nitrogen (NH_3—_N) concentration, and VFA are summarized in Table 5. Rumen pH was not significantly affected by the treatments. Apricot biochar significantly reduced NH_3—_N compared to other treatments. For protozoa population, no significant differences were observed between the control and walnut biochar, but apricot and almond biochars significantly reduced protozoa numbers compared to the other treatments (P < 0.05). Ruminal VFA profiles were evaluated in two units: absolute concentration (mmol/L) and molar proportion (mmol/100 mol). Regarding the total fermentative capacity (mmol/L), the walnut biochar treatment significantly (P < 0.05) increased the total VFA concentration to 111 mmol/L, compared to 102 mmol/L in the control group. In contrast, almond and apricot shell biochars reduced total VFA concentrations to 85.7 and 74.5 mmol/L, respectively. Walnut shell biochar also resulted in the highest propionate concentration (26.2 mmol/L). In terms of molar proportions (mmol/100 mol), which reflect shifts in microbial metabolic pathways, the control group exhibited the highest acetate percentage (67%). However, all biochar treatments (walnut, almond, and apricot) significantly increased the molar proportion of propionate (up to 25.6%) and decreased the acetate-to-propionate (A/P) ratio. The lowest A/P ratios were recorded in the almond (2.14), apricot (2.18), and walnut (2.52) treatments, compared to 3.42 in the control, indicating a significant redirection of fermentation pathways away from acetate toward propionate production.

**Table 5:** Effect of experimental treatments on rumen fermentation parameters.

Experimental treatments*						
Parameters	Con	Con+WS-B	Con+Als-B	Con+Aps-B	SEM	*p*-value
NH_3—_N (mg/dL)	13.2^a^	15.7^a^	13.5^a^	8.11^b^	0.98	0.0036
pH	6.22	6.22	6.24	6.24	0.02	0.8484
Protozoa ($10^{4}/\text{mL}$)	9.66^a^	8.5^a^	7.08^b^	6.83^b^	0.39	0.0031
*Total VFA (mmol/L)*	102^b^	111^a^	85.7^c^	74.5^d^	2.23	<0.001
Acetate	68.6^a^	66.2^a^	47.1^b^	39.3^c^	2.23	<0.001
Propionate	20^bc^	26.2^a^	21.9^b^	18.1^c^	0.68	0.0002
Butyrate	10.9^b^	14.6^a^	13.2^a^	14.4^a^	0.66	0.0155
Iso-Butyrate	0.44^bc^	0.64^a^	0.54^ab^	0.38^c^	0.03	0.0039
Valerate	1.69	2.02	2.09	1.68	0.21	0.4273
Iso-Valerate	0.66^b^	0.92^a^	0.81^a^	0.67^b^	0.03	0.0025
Acetate/propionate	3.42^a^	2.52^b^	2.14^c^	2.18^c^	0.09	<0.001
*Total VFA (mmol/100mol)*						
Acetate	67.0^a^	59.8^b^	55.0^c^	52.7^c^	1.21	0.0002
Propionate	19.5^b^	23.8^a^	25.6^a^	24.2^a^	0.55	0.0003
Butyrate	10.7^c^	13.2^bc^	15.4^b^	19.4^a^	0.83	0.0005
Iso-Butyrate	0.44^b^	0.58^a^	0.63^a^	0.51^ab^	0.03	0.0306
Valerate	1.70	1.82	2.44	2.24	0.23	0.1454
Iso-Valerate	0.64^b^	0.83^a^	0.94^a^	0.90^a^	0.03	0.0030

⁎ Experimental diets contained without biochar as a control (CON), walnut shell biochar (WS-B 1%), almond shell biochar (Als-B 1%), and apricot shell biochar (Aps-B 1%) (n = 3). Values within a row with different superscripts differ significantly at p < 0.05.

### 3.4 Methane production

**Fig. 1:**
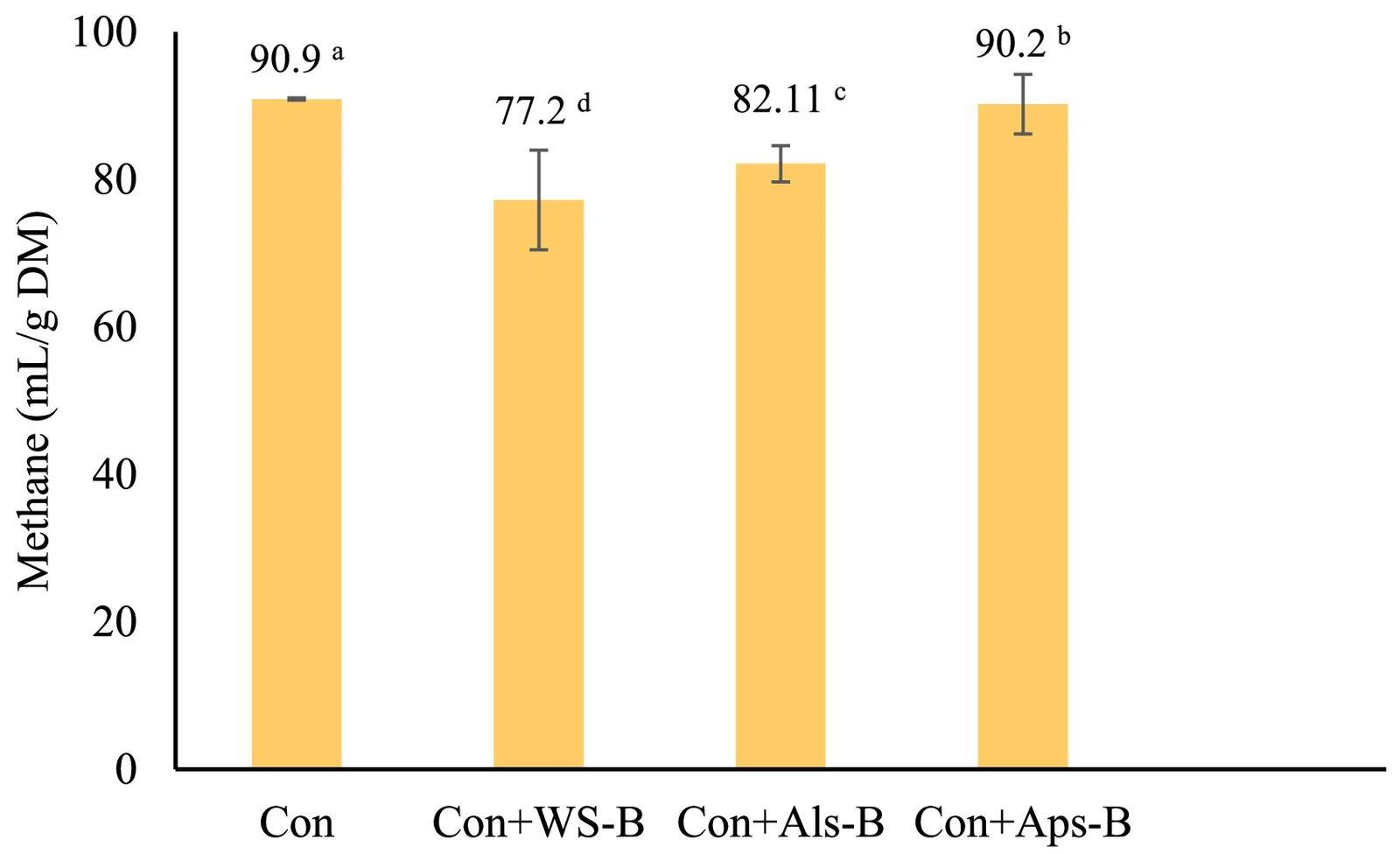
Effect of experimental treatments on methane emission measured by *in vitro* technique.

As shown in Fig. 1, CH_4_ production (mL/g DM) showed a significant reduction in all biochar treatments compared to the control. Notably, the highest CH_4_ production (90.9 mL/g DM) was observed in the control treatment, while the lowest (77.2 mL/g DM) was recorded for walnut biochar (P < 0.05).

Experimental diets included: control without biochar (CON), walnut shell biochar (WS-B), almond shell biochar (Als-B), and apricot shell biochar (Aps-B) (n = 3). ^a,b,c^ Values with different superscripts above the bars indicate significant differences at p < 0.05 (SEM = 0.09)

### 3.5 In vitro dry matter degradability

The results of *in vitro* DMD for the control and control with biochars derived from walnut, almond, and apricot are presented in Table 4. The results indicated that walnut biochar significantly increased *in vitro* ruminal DMD (72%) compared to control and other treatments (P < 0.05). However, total tract degradability did not differ significantly among almond, walnut, and control treatments, all showing similar values between 74 and 76%, whereas apricot biochar exhibited significantly lower total tract degradability at 71% (P < 0.05).

## 4 Discussion

### 4.1 Biochar yield, elemental analysis, and BET surface area

The yield of biochar derived from lignocellulosic biomass during pyrolysis can be influenced by various factors, including the content of cellulose, hemicellulose, and lignin, the interactions among these components, their inherent structures and chemical properties, and biomass structure (*e.g.*, size and density) (Lehmann et al., 2011). During pyrolysis, dehydration, cleavage, and polymerization reactions lead to the decomposition and reformation of C compounds, while other elements may be lost due to volatilization. Consequently, the total C content of biochar increases with the pyrolysis temperature (Weber & Quicker, 2018).

Understanding the ratios of H, C, and O can provide insights into the biochar's longevity. Specifically, the O/C and H/C ratios have been used to characterize the carbonization process of pyrolysis and key factors influencing biochar's environmental durability as a function of these ratios (Spokas, 2010). The lower O/C ratio of almond biochar (0.03) compared to walnut and apricot biochars (0.12 and 0.21, respectively) indicates the greater stability of almond biochar, which is a significant advantage for carbon sequestration.

In recent years, significant efforts have been made by organizations such as the International Biochar Initiative and the European Biochar Certificate to standardize biochar production and safety assessment. According to these standards, the H/C and O/C molar ratios in biochar should not exceed 0.7 and 0.4, respectively, to ensure product safety, particularly for applications in animal feed. Furthermore, the use of natural, untreated biomass as a feedstock is strongly emphasized (EBC, 2012; IBI, 2015). The findings of this study demonstrate that the biochars derived from walnut, almond, and apricot shells possess H/C and O/C ratios well below these critical thresholds and are produced from natural, uncontaminated feedstocks. Consequently, based on these standardized criteria, the produced biochars can be considered safe for inclusion in animal nutrition.

Hemicellulose and cellulose decompose in the temperature range of 200 to 400 °C, while lignin has a more complex structure and degradation mechanisms. The decomposition of lignin primarily occurs in the temperature range of 350–500 °C and continues up to 900 °C. Therefore, the significant increase in surface area and pore volume in the nanometer range mainly occurs in the 300–500 °C range (Ma et al., 2019). It has been reported that the development of nanometer sized pores during pyrolysis is closely related to the chemical structure and lignin content of the raw biomass. The higher the lignin content in the biomass, the greater the surface area and pore volume in the nanometer range when produced through slow pyrolysis in the 350 to 600 °C range (Fu et al., 2012).

Since the biochars in this study were produced under identical thermal conditions, the differences in their characteristics are mainly attributed to the initial composition of the biomass. Therefore, it is likely that the higher lignin content in walnut shell may be the main factor responsible for the increased specific surface area observed in the resulting biochar.

### 4.2 Gas production, gas production kinetic, and estimated parameters

Gas production during ruminal fermentation is commonly used to evaluate fiber digestion kinetics under laboratory conditions. Biochar's strong adsorption properties, stemming from its association with organic compounds, may influence gas production. Increased gas production could reflect a stimulatory effect of biochar on rumen microbial activity and fermentation. One possible explanation is that biochar may enhance microbial growth efficiency by facilitating closer interactions between microbial colonies, thereby improving ATP production and utilization efficiency (Qomariyah et al., 2023). However, as microbial community dynamics were not analyzed in the present study, these mechanisms remain hypothetical and require confirmation through further microbiological investigations.

In this study, the source of biochar significantly influenced total gas production. Walnut shell biochar increased total gas production compared to the control, while apricot shell biochar exhibited the opposite effect. In the case of apricot shell biochar, the reduced gas production may be associated with a shift in ruminal fermentation pathways, whereby fermentable substrates are directed less toward gaseous end products. Such a shift could be linked to greater allocation of metabolic energy toward microbial biomass synthesis rather than gas formation. Similarly, Van Dung et al. (2024b) observed that corn stalk biochar produced more gas than biochars from rice straw and bamboo, with variations linked to pyrolysis temperatures (300, 500, and 700 °C). Cabeza et al. (2018) reported no significant differences in gas production between biochars derived from five biomass sources (miscanthus straw, rapeseed straw, rice bran, softwood pellets, and wheat straw) across two pyrolysis temperatures (550 °C and 700 °C), although slightly higher gas production was noted at 700 °C. Other studies (McFarlane et al., 2017; Saleem et al., 2018) similarly observed no notable effects of biochar on total gas production.

### 4.3 Rumen ecosystem

Ammonia is the primary nitrogen source for microbial protein synthesis in the rumen and represents the final product of dietary protein degradation by rumen microbes (Reynal et al., 2007). Within the rumen, dietary proteins are hydrolyzed and deaminated to yield free peptides and ammonia, which are subsequently utilized by rumen microbes for growth. Elevated ammonia concentrations promote microbial protein synthesis, as ammonia is a key precursor for microbial cell formation. The impact of biochar on NH_3—_N production is variable, with studies reporting no effect (Pereira et al., 2014), reductions (Cabeza et al., 2018), or even increases (Saleem et al., 2018).

Since *in vitro* incubation occurs within a closed system, two potential mechanisms could explain the observed differences in NH_3—_N concentrations. These differences may result from either a reduction in the proteolysis and deamination of nitrogenous substrates or an increase in the utilization of NH_3—_N for microbial protein synthesis, or a combination of both processes. Although NH_3_-N concentration was numerically higher in the walnut biochar treatment than in the control, the difference was not statistically significant. This aligns with the increased production of branched-chain VFAs observed in this study, which may indicate changes in amino acid fermentation. However, further research is necessary to validate this hypothesis, as protein metabolism was beyond the scope of the present study. In this context, the addition of walnut biochar in the study by Mirheidari et al. (2020) and biochar derived from agricultural residues and potato peels in Rodrigues et al. (2020) was also found to increase NH_3—_N concentrations. Variations in biochar sources, driven by differences in their structure and composition, can result in diverse effects on rumen fermentation.

In the present study, biochars had no effect on rumen pH, consistent with findings from Lind et al. (2024) and Qomariyah et al. (2021). The current study demonstrated that biochar application reduced protozoal populations, although the extent of this reduction varied among treatments. The reduction in protozoal populations observed with almond and apricot shell biochars may be related to differences in the structure and composition of the biomass sources used for biochar production, which could lead to differential responses in rumen fermentation (Saleem et al., 2018). Walnut shell biochar, however, did not significantly reduce protozoa, which is consistent with previous findings reporting no protozoal reduction with walnut-derived biochar (Garillo et al., 1994; Tamayao et al., 2021). Methane production showed a partially similar pattern: the reduction in CH_4_ observed with almond shell biochar corresponded with the decline in protozoal populations, given their established role in hydrogen transfer to methanogens (Mosoni et al., 2011). However, this association should be interpreted with caution, as methanogen populations were not directly quantified and a causal relationship cannot be confirmed. In the apricot shell biochar, however, the numerical reduction in CH_4_ was very small and therefore not biologically meaningful despite reaching statistical significance. Thus, the substantial decrease in protozoal counts in the apricot treatment did not translate into a meaningful reduction in CH_4_ production, suggesting that CH_4_ formation is influenced by multiple factors beyond protozoal abundance alone (Guyader et al., 2014). Furthermore, because walnut shell biochar markedly reduced CH_4_ without significantly affecting protozoal counts, its CH_4_ lowering effect cannot be attributed to protozoal reduction and is likely driven by other biochar related mechanisms (Saleem et al., 2018). Overall, although some treatment effects were statistically significant, their practical biological relevance should be interpreted with caution when the numerical differences were relatively small.

Volatile fatty acids are the primary end products of rumen fermentation, providing approximately 80% of the energy requirements for ruminants. Saleem et al. (2018) associated increased VFA production with enhanced degradability of DM, organic matter, crude protein, and fiber. Biochar's proposed role as an electron mediator in redox reactions during digestion (Teoh et al., 2019) may improve extracellular hydrogen transfer within rumen microbial populations, potentially enhancing microbial enzyme activity and fostering synergistic interactions among microbial species involved in VFA synthesis.

Although walnut shell biochar increased total VFA concentration, almond and particularly apricot shell biochars reduced total VFA compared with the control. This reduction may indicate lower overall ruminal fermentation activity and suggests that the effects of biochar depend on the physicochemical properties of the feedstock, which can differentially influence microbial activity and substrate fermentation (McFarlane et al., 2017). Despite these differences in total VFA concentration, all biochar treatments consistently reduced the acetate-to-propionate (A:P) ratio compared to the control (Table 5).

A lower A:P ratio is a desirable outcome in rumen fermentation, as it enhances energy efficiency. The energy efficiency of glucose fermentation into acetate, propionate, and butyrate is 62%, 109%, and 78%, respectively (Li et al., 2018). Propionate is the most energy efficient fermentation product due to its role in hydrogen absorption and as a primary precursor for gluconeogenesis (Li et al., 2018). A reduced A:P ratio decreases hydrogen release and limits hydrogen availability for CH_4_ production (Demirtas et al., 2023). Thus, the effect of biochars on the A:P ratio may partially explain the observed reduction in CH_4_ production. Although all biochars lowered the A:P ratio, CH_4_ did not decrease in the same way in all treatments, suggesting that other factors may also be involved. In a study by Mirheidari et al. (2020), biochar had no effect on total VFA production, while McFarlane et al. (2017) similarly reported no changes in VFA production with biochar addition under *in vitro* conditions.

### 4.4 Methane production

The livestock breeding is a major contributor to CH_4_ emissions, as ruminant animals generate significant amounts of CH_4_ during digestion. Methane emissions from anaerobic biological sources are the result of a balance between production by methanogenic archaea and oxidation by methanotrophic microorganisms (Knittel & Boetius, 2009). Methanotrophs oxidize CH_4_ to derive energy, utilizing various electron acceptors under both aerobic and anaerobic conditions. Initially, methanotrophy was considered exclusively oxygen dependent, with aerobic methanotrophs long thought to represent the only biological pathway for CH_4_ oxidation. These aerobic methanotrophs were classified entirely within the phylum *Proteobacteria*. However, discoveries over the past two decades have broadened this perspective, revealing methanotrophic microorganisms outside the *Proteobacteria* phylum and even archaea capable of anaerobic CH_4_ oxidation using alternative electron acceptors such as sulfate, nitrate, iron, and manganese (Guerrero-Cruz et al., 2021). Although this study did not quantify microbial populations, it is hypothesized that the observed reduction in CH_4_ production may be linked to the potential activity of such methanotrophic populations. Nevertheless, this remains a speculative mechanism that warrants further molecular investigation to confirm the presence and contribution of these microorganisms to CH_4_ mitigation in the rumen.

When biochar is added to ruminant feed, it may modify the microbial population. Biochars with large surface areas and highly porous structures could create a favorable habitat for methanotrophic microorganisms, which may play a crucial role in the methanogen methanotroph interaction, thereby enhancing anaerobic CH_4_ oxidation potential (Leng et al., 2012). It is hypothesized that biochar might increase the ratio of methanotrophs to methanogens, leading to a reduction in CH_4_ production. In the present study, the BET surface area of the biochars was determined to be 618, 178, and 101 m²/g for walnut, almond, and apricot shell biochars, respectively (Table 2). While the greatest reduction in CH_4_ production was observed with walnut shell biochar, which also exhibited the highest surface area, further research is required to definitively link these physical characteristics to changes in the microbial community.

In addition, the reduction in CH_4_ production may also be attributed to secondary compounds present in biochar, such as phenolic compounds. Phenolic compounds may inhibit CH_4_ production by potentially disrupting the cell walls of methanogenic bacteria, thereby possibly inhibiting their growth (Jayanegara et al., 2015). During the pyrolysis process, lignin and cellulose may not be completely decomposed, potentially leaving behind phenolic compounds. Typically, water soluble phenols (phenolic compounds that dissolve in water or polar solvents) might be degraded, while other phenolic compounds may remain intact.

Leng et al. (2012) reported a 24% reduction in CH_4_ production in cattle fed rice husk biochar at a 0.62% inclusion rate of the diet. Winders et al. (2019) observed numeric reductions of 9.5% and 18.4% in CH_4_ production (g/kg DMI) when pine biochar (0.8% inclusion) was added to forage based and concentrate based diets, respectively. In the study by Van Dung et al. (2024a), rice husk biochar at 1%, 3%, 5%, and 7% inclusion levels (DM) significantly reduced CH_4_ production under *in vitro* conditions. However, studies by Pereira et al. (2014), O’Reilly et al. (2021), and Tamayao et al. (2021) found no impact of biochar inclusion on CH_4_ production. The variable results regarding biochar’s efficacy in reducing CH_4_ may be attributed to differences in biochar properties, such as particle size, surface area, electrical conductivity, adsorption capacity, pyrolysis temperature, and application rates (Teoh et al., 2019).

### 4.5 *In vitro* dry matter degradability

Nutrient degradability is closely linked to CH_4_ production in ruminants, as improved degradation reduces the substrates available for methanogenic microbes. Enhanced nutrient degradability, particularly from fibrous feeds, decreases the amount of undigested material entering the intestines, where CH_4_ is also produced. Improved degradation can lead to shifts in ruminal fermentation, potentially reducing CH_4_ emissions (Konkova & Halmemies-Beauchet-Filleau, 2024). For instance, Janssen (2010) highlighted that increased feed digestibility can lower CH_4_ yield per unit of digested material, making nutrient digestibility a crucial factor in mitigating enteric CH_4_ emissions from ruminants.

This positive response can be attributed to the porous structure of biochar, which provides a suitable habitat for microbial colonization and promotes biofilm formation (Leng et al., 2013). Enhanced activity of fibrolytic bacteria due to increased biofilm formation can improve substrate degradation efficiency. In the present study, biochars with high porosity and surface area facilitated greater biofilm development, resulting in improved DMD. Consistent with the total gas production results, walnut biochar, which showed the highest gas production, also exhibited the highest DMD.

Overall, the differential responses observed among the evaluated biochar sources suggest that their effects on ruminal fermentation and CH_4_ production are highly dependent on feedstock characteristics. These findings underscore the importance of careful selection and characterization of biochar materials prior to their application in ruminant nutrition. From a practical standpoint, identifying biochar sources with greater mitigation potential may support the development of more targeted and efficient CH_4_ reduction strategies in ruminant production systems. Nevertheless, further *in vivo* studies are required to confirm these effects under practical feeding conditions.

## 5 Conclusions

This study demonstrated that different types of biochar exert varying effects on rumen fermentation parameters under *in vitro* conditions. Among the biochars tested, walnut shell biochar showed the greatest reduction in CH_4_ production under these conditions. Walnut shell biochar also enhanced *in vitro* OMD and increased total VFA and propionate concentrations, whereas almond and apricot shell biochars reduced *in vitro* DMD. However, further studies, especially *in vivo* experiments, are necessary for more accurate evaluation and practical application of these findings.

## Limitations

This study has several limitations that should be considered when interpreting the results. The experiment was conducted entirely under *in vitro* conditions, and the use of rumen fluid from only two donor animals and a single biochar inclusion level limits the generalizability of the findings. In addition, all incubations were conducted in a single experimental run using pooled rumen inoculum, and the *in vitro* degradability assay was performed with multiple nylon bags incubated in a single digestion bottle. Furthermore, a comprehensive analysis of microbial communities including cellulolytic, proteolytic, methanotrophs, and methanogens is essential for a deeper understanding of rumen fermentation. Due to financial constraints and the lack of access to advanced laboratory equipment and molecular techniques such as qPCR or sequencing, such detailed microbial analyses could not be performed in this study. Therefore, the proposed microbial and fermentation mechanisms remain hypothetical, and future *in vivo* studies employing advanced molecular approaches are recommended to validate the present findings.

## Ethics declaration

This study was conducted in accordance with the ARRIVE (Animal Research: Reporting of *In Vivo* Experiments) guidelines. This study was approved by the University of Tabriz. (Approval No. IR.TABRIZU.REC.1403.129)

## CRediT authorship contribution statement

**Mehri Montazerharzand:** Writing – review & editing, Writing – original draft, Methodology, Formal analysis, Data curation. **Hamid Paya:** Writing – review & editing, Supervision, Methodology, Data curation, Conceptualization. **Akbar Taghizadeh:** Writing – review & editing, Formal analysis. **Ali Hosseinkhani:** Writing – review & editing, Methodology, Formal analysis. **Mohammad Ramin:** Writing – review & editing, Validation, Supervision, Methodology, Data curation, Conceptualization.

## Declaration of competing interest

The authors declare that they have no known competing financial interests or personal relationships that could have appeared to influence the work reported in this paper.
